# NAFLD indirectly impairs antigen-specific CD8^+^ T cell immunity against liver cancer in mice

**DOI:** 10.1016/j.isci.2022.103847

**Published:** 2022-02-01

**Authors:** John C. McVey, Benjamin L. Green, Benjamin Ruf, Justin D. McCallen, Simon Wabitsch, Varun Subramanyam, Laurence P. Diggs, Bernd Heinrich, Tim F. Greten, Chi Ma

**Affiliations:** 1 Gastrointestinal and Thoracic Malignancy Section, National Cancer Institute, National Institutes of Health, TGMB NIH/NCI/CCR Building 10 Rm 3B44 9000 Rockville Pike, Bethesda, MD, USA; 2 Department of Surgery, Perelman School of Medicine, University of Pennsylvania, Philadelphia, PA, USA; 3 Surgical Oncology Program, Center for Cancer Research, National Cancer Institute, National Institutes of Health, Bethesda, MD, USA; 4 Brody School of Medicine, East Carolina University, Greenville, NC, USA; 5 Department of Surgery, Rutgers New Jersey Medical School, Newark, NJ, USA; 6 Department of Medicine, Hannover Medical School, Hannover, Germany; 7 NCI CCR Liver Cancer Program, National Institutes of Health, Bethesda, MD, USA

**Keywords:** Immunology, Cancer

## Abstract

Non-alcoholic fatty liver disease (NAFLD) has become an important etiology leading to liver cancer. NAFLD alters adaptive T cell immunity and has a profound influence on liver cancer development. However, it is unclear how NAFLD affects tumor antigen-specific T cell response. In this study, we generated a doxycycline-inducible MHC-I and -II antigen-expressing HCC cell line which allowed us to investigate tumor antigen-specific T cell response in two NAFLD mouse models. The system proved to be an effective and efficient way to study tumor antigen-specific T cells. Using this model, it was found that NAFLD impairs antigen-specific CD8^+^ T cell immunity against HCC. The effect was not due to reduced generation or intrinsic functional changes of tumor antigen-specific CD8^+^ T cells but caused by accumulated macrophages in the liver environment. The findings suggest that targeting macrophages in NAFLD-driven HCC may improve therapeutic outcomes.

## Highlights

• The creation of a novel doxycycline-inducible antigen presenting HCC mouse model
• Diet and genetic NAFLD mice have impaired TAS CD8 T cell response to HCC
• NAFLD does not change the intrinsic function of TAS CD8 T cells
• Depletion of macrophages reverses the immunosuppressive environment in NAFLD mice

## Graphical abstract

**Figure:**
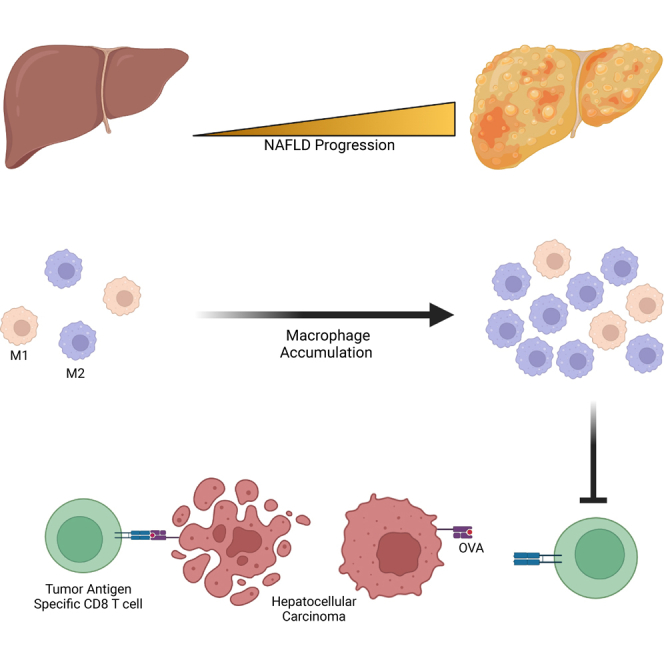

## Introduction

Liver cancer is a leading cause of cancer-related deaths. Hepatocellular carcinoma (HCC, ∼80%) and cholangiocarcinoma (∼10%–20%) make up most of primary liver cancers and have become a growing health challenge worldwide (Llovet et al., 2021). Patients with liver cancer are often diagnosed with unresectable disease, resulting in a dismal prognosis due to the low efficacy of current treatments (Forner et al., 2018; Llovet et al., 2008). Non-alcoholic fatty liver disease (NAFLD) has been established as an important risk factor for liver cancer. NAFLD comprises a spectrum of liver pathologies with excessive hepatic lipid accumulation ranging from simple steatosis to more severe forms of non-alcoholic steatohepatitis (NASH) with histological necroinflammation. NAFLD is strongly associated with obesity and following the increasing prevalence of obesity, the incidence of NAFLD-induced HCC is rising (Anstee et al., 2019).

NAFLD has profound impacts on the hepatic local adaptive immune system, which has been found to play a critical role in controlling liver cancer. In NAFLD animal models, the accumulation of activated IgA-producing B cells, and Th17 cells have been shown to promote HCC (Shalapour et al., 2017, Ana et al., 2016). Another study demonstrated that loss of liver CD4^+^ T cells also contributes to NAFLD-accelerated HCC (Ma et al., 2016). CD8^+^ T cells are crucial for anti-tumor immunity and success of tumor immunotherapy. The NAFLD-caused CD8^+^ T cell activation has been repeatedly reported, however, their role in NAFLD-promoted HCC is controversial. In a model of NASH-induced HCC using choline-deficient high-fat diet (CD-HFD), CD8^+^ T cells were found to promote NASH disease progression and subsequent HCC development. The pro-NASH function of CD8^+^ T cells was supported by sc-RNA-seq studies which identified that NASH causes hepatic accumulation of auto-aggressive CXCR6^+^ CD8^+^ T cells to kill hepatocytes in an MHC-I-independent manner (Dudek et al., 2021). Importantly, in both meta-analysis of patients with HCC and the NASH-induced HCC CD-HFD mouse model, anti-PD1 therapy lost efficacy in the context of NASH (Pfister et al., 2021). In contrast, in the HFD-fed MUP-uPA HCC mouse model, removing CD8^+^ T cells accelerated HCC, suggesting that CD8^+^ T cells have anti-HCC function in NASH. In addition, reenergizing CD8^+^ T cells using anti-PD-L1 antibody reduced HCC in NASH mice (Shalapour et al., 2017). All these reports show that further investigations are needed to clarify the roles of immune cells in HCC regulation in NAFLD.

Gene mutations are commonly present in tumors, and the resultant neoantigens allow immune cells to distinguish the tumor from the host (Chalabi et al., 2020; DuPage et al., 2011; Mandal et al., 2019). Tumor antigen-specific (TAS) T cells are one of the primary immune cell subsets that provide anti-tumor immunity in HCC and metastatic liver cancer (Flecken et al., 2014; Lu et al., 2021; Paczesny et al., 2004; Reissfelder et al., 2015). TAS T cells are also key players in many immune-based therapies such as the novel checkpoint inhibitors recently approved for HCC. Although critical for tumor controlling, TAS T cells often comprise only a small part of total T cells. Even in the tumor-infiltrating lymphocytes, most of T cells do not recognize tumor antigens (Li et al., 2021). Importantly, total T cell analyses even using sc-RNA-seq alone are often not sensitive enough to identify the changes of TAS T cells (Caushi et al., 2021). Peptide-MHC multimers have become the standard for detection of TAS T cells recognizing known antigens. Until now, there has been little investigation into how NAFLD affects TAS T cells.

To better delineate the changes NAFLD induces on TAS T cells, we generated a mouse HCC cancer cell line that contained inducible MHC class I- and II-restricted chicken ovalbumin (OVA) antigens. The cell line was then seeded into NAFLD livers, and OVA-specific T cells were interrogated to characterize changes in their quantity and function. Further investigation into the immune environment of NAFLD livers was also done to determine its interaction with TAS T cells.

## Results

### Generating an inducible OVA antigens-expressing HCC mouse model to study tumor antigen-specific CD8^+^ T cell response

**Figure 1:**
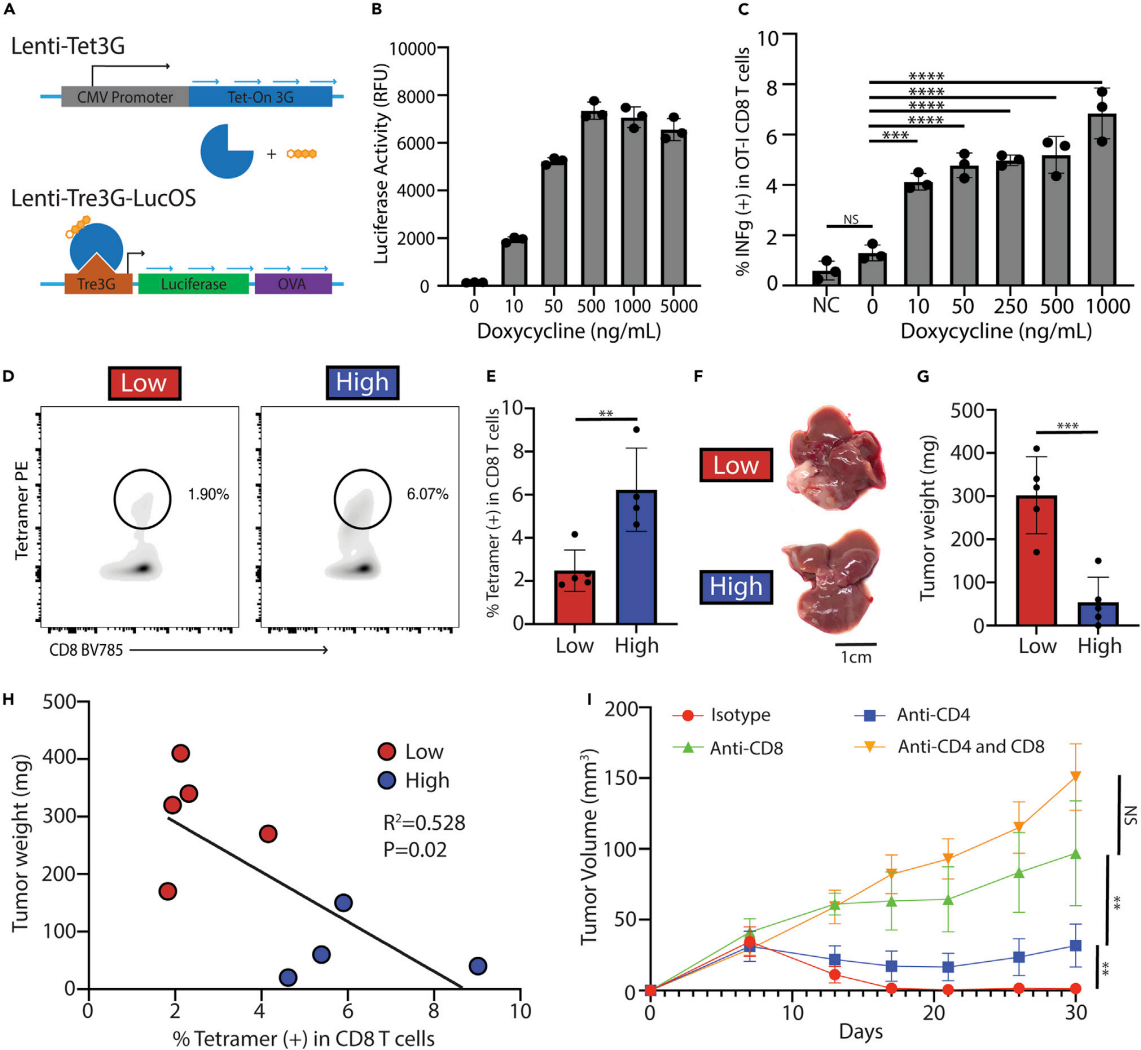
Creation of an inducible LucOS-expressing mouse HCC cell line (A) Diagram showing how doxycycline induces the TetOn system to express luciferase and OVA MHC-I and -II antigens (LucOS). Blue arrows represent turning on of genes. (B) Luciferase activity of the Hep55.1c cell line transduced with the Tet3G-LucOS expression system incubated with increasing concentrations of doxycycline. (C) Intracellular IFNg production in OT-I CD8^+^ T cells co-incubated with Hep55.1c-Tet3G-LucOS cells as well as doxycycline and IFNg (10 ng/mL). NC means negative control and was Hep55.1c cells not transduced with the Tet3G-LucOS system. (D) FACs plot showing OVA_257-264_ tetramer-positive CD8^+^ T cells from liver lymphocytes of mice treated with dox water or control water. (E) Bar graph of (D) (F and G) Representative picture and (G) tumor weight of mouse livers or tumors 10 days after an intrahepatic injection of Hep55.1c-Tet3G-LucOS given normal or doxycycline water. Low means low antigen expression due to no dox water while high means high antigen expression due to dox water. (H) Correlation of tumor weight and percentage of tetramer-positive CD8^+^ T cells. (I) Growth curves of flank Hep55.1c-Tet3G-LucOS tumors in mice given doxycycline water and depleted for CD4^+^ and/or CD8^+^ T cells (n = 5 per group). All pooled data presented as mean with SD. One-way ANOVA with Tukey correction for (B), (C) & (I) and unpaired t test for (E) & (G) ∗p < 0.05; ∗∗p < 0.01; ∗∗∗p < 0.001; ∗∗∗∗p < 0.0001.

A previous study demonstrated that HCC tumor expression of MHC-I and –II-restricted OVA antigens using LucOS, a construct containing the MHC-I OVA_257-264_ and MHC-II _OVA329-337_ antigens conjugated to luciferase, caused a strong T cell-dependent anti-tumor response (Ruiz de Galarreta et al., 2019). Also, considering the availability of tetramers to detect these OVA antigen-specific T cells, we planned to study TAS T cells using OVA-antigen expressing HCC tumors. An inducible LucOS construct was generated using the commercially available Tet3G doxycycline (dox)-regulated expression system (Figure 1A). To generate an HCC cell line-expressing Tet3G-LucOS, the construct was transduced using lentivirus into the Hep55.1c mouse HCC cell line (collectively called Hep55.1c-Tet3G-LucOS). The inducible expression of OVA antigens in Hep55.1c-Tet3G-LucOS was demonstrated *in vitro* by detecting increased luciferase activity in a dox dose-dependent manner (Figure 1B). Next, we tested whether OVA antigen-expressing Hep55.1c-Tet3G-LucOS tumor cells can be recognized by OT-I CD8^*+*^ T cells, which express transgenic TCR-recognizing OVA_257-264_ peptide-MHC-I complex. Indeed, following increasing concentrations of dox, more INFg production was observed in cocultured OT-I CD8^*+*^ T cells (Figure 1C). It was also found that the *in vitro* activation of OT-I CD8^+^ T cells by Hep55.1c-Tet3G-LucOS tumor cells was dependent on the addition of IFNg due to low basal expression of tumor surface H-2K^b^ (MHC-I) (Figures S1A–S1D).

To confirm the system's function *in vivo*, the Hep55.1c-Tet3G-LucOS cells were seeded into the livers of C57Bl/6 mice. Tumor OVA antigen expression was induced by feeding mice with dox (2 mg/mL) supplemented in the drinking water. Flow cytometry analysis of lymphocytes isolated from the tumor-bearing livers was used to detect the OVA_257-264_-specific CD8^+^ T cells using H-2Kb/OVA_257-264_ tetramer. A low level of OVA_257-264_-specific CD8^+^ T cells was detected in tumor-bearing mice even without induction by dox, which is absent in tumor-free mice (data not shown). This allowed us to compare OVA antigen high-expressing (OVA^high^) tumors with OVA antigen low-expressing (OVA^low^) tumors (Figures 1D and 1E). Indeed, more OVA_257-264_-specific CD8^+^ T cells were found in OVA^high^ tumor-bearing mice (Low: 1.44% vs High: 6.33%, p < 0.01. Figures 1D and 1E). As expected, a drastic decrease in tumor weight was observed in OVA^high^ tumors compared with OVA^low^ tumors (Low: 302mg vs High: 54mg, p < 0.001) (Figures 1F and 1G). An inverse correlation was found between the OVA_257-264_-specific CD8^+^ T cells and tumor weight (R^2^ = 0.582 and p = 0.02) (Figure 1H) suggesting their important role in tumor controlling. In contrast, the level of total CD8^+^ T cells did not change between mice bearing OVA^high^ or OVA^low^ tumors (Figure S1E). Next, we tested the contribution of T cells to the OVA expression-caused tumor inhibition. Hep55.1c-Tet3G-LucOS cells were injected into the flanks of mice given dox water, then depletion of CD4^+^, CD8^+^, or CD4^+^ and CD8^+^ T cells. OVA expression caused a complete tumor regression. Consistent with a previous report (Ruiz de Galarreta et al., 2019), removing CD4^+^ and CD8^+^ T cells reversed the tumor suppression in OVA-expressing HCC tumors, and the effect was mostly mediated by CD8^+^ T cells (Figure 1I). The results confirm the successful creation of an inducible OVA antigen-expressing HCC tumor model which can generate a strong anti-tumor CD8^+^ T cell response. This model enables us to study the regulation of tumor antigen-specific CD8^+^ T cell response by assessing the OVA_257-264_-specific CD8^+^ T cells as a surrogate.

### NAFLD impairs antigen-specific anti-tumor immunity against liver tumor

**Figure 2:**
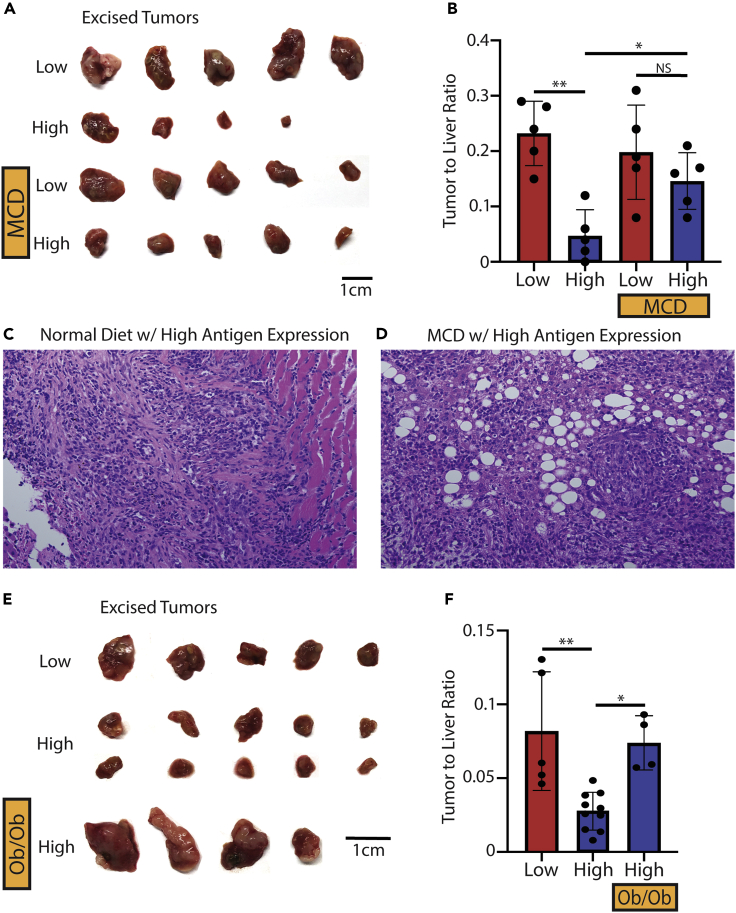
Intrahepatic injection of Hep55.1c-Tet3G-LucOS cells in normal or NAFLD livers (A–D) Excised tumors and (B) tumor to liver ratio of mice fed normal (ND) or a methionine-choline-deficient (MCD) diet with or without doxycycline (dox) 10 days after an intrahepatic injection of Hep55.1c-Tet3G-LucOS cells. Low means low antigen expression due to no dox water while high means high antigen expression due to dox water. Representative histochemistry of (C) ND with dox and (D) MCD with dox livers from mice bearing Hep55.1c-Tet3G-LucOS tumors. All images were taken at |×10×| magnification. (E and F) Excised tumors and (F) tumor to liver ratio of normal C57BL/6 or Ob/Ob mice given normal or doxycycline water 10 days after an intrahepatic injection of Hep55.1c-Tet3G-LucOS cells. All pooled data presented as mean with SD. One-way ANOVA with Tukey correction for A and (B) ∗p < 0.05; ∗∗p < 0.01; ∗∗∗p < 0.001; ∗∗∗∗p < 0.0001.

A growing body of evidence has suggested that adaptive immunity plays a critical role in the regulation of NAFLD-promoted liver cancer (Heinrich et al., 2021; Ma et al., 2016; Shalapour et al., 2017; Dudek et al., 2021; Cai et al., 2019; Pfister et al., 2021). Next, we investigated the influence of NAFLD on tumor antigen-specific CD8^+^ T cell response using our newly generated mouse model. The Hep55.1c-Tet3G-LucOS tumors were seeded in the livers of NAFLD mice fed with MCD diet or control mice given normal diet, and OVA expression was controlled by feeding mice with or without dox water. As expected, in mice given normal diet, liver OVA^high^ tumors were significantly smaller compared with OVA^low^ tumors (ND low: 0.23 vs ND high: 0.05, p = 0.001) (Figures 2A and 2B). Interestingly, in NAFLD mice, tumor OVA expression failed to affect the growth of liver tumors (MCD low: 0.20 vs MCD high: 0.15, p = 0.56) (Figures 2A and 2B). In fact, after inducing tumor OVA expression, the NAFLD mice had a larger tumor burden compared to the normal diet-fed mice (ND low: 0.05 vs MCD high: 0.15, p < 0.05). The presence of viable liver tumor was confirmed (Figures 2C and 2D).

To confirm these findings, the experiment was repeated in a second genetic-induced NAFLD model using Ob mice (Trak-Smayra et al., 2011; Brown et al., 2018c). Similarly, high tumor OVA expression caused a strong decrease of liver tumor in control lean mice (0.08 vs 0.02, respectively, p < 0.01). Again, liver OVA^high^ tumors were larger in Ob NAFLD mice compared to that in control lean mice (0.07 vs 0.02 respectively, p = 0.03) (Figures 2E and 2F). Together, these results demonstrate that NAFLD impairs TAS anti-tumor immunity against liver tumor even in a highly immunogenic tumor expressing OVA antigens.

### NAFLD has no clear inhibition on generation or function of tumor antigen-specific CD8^+^ T cells

**Figure 3:**
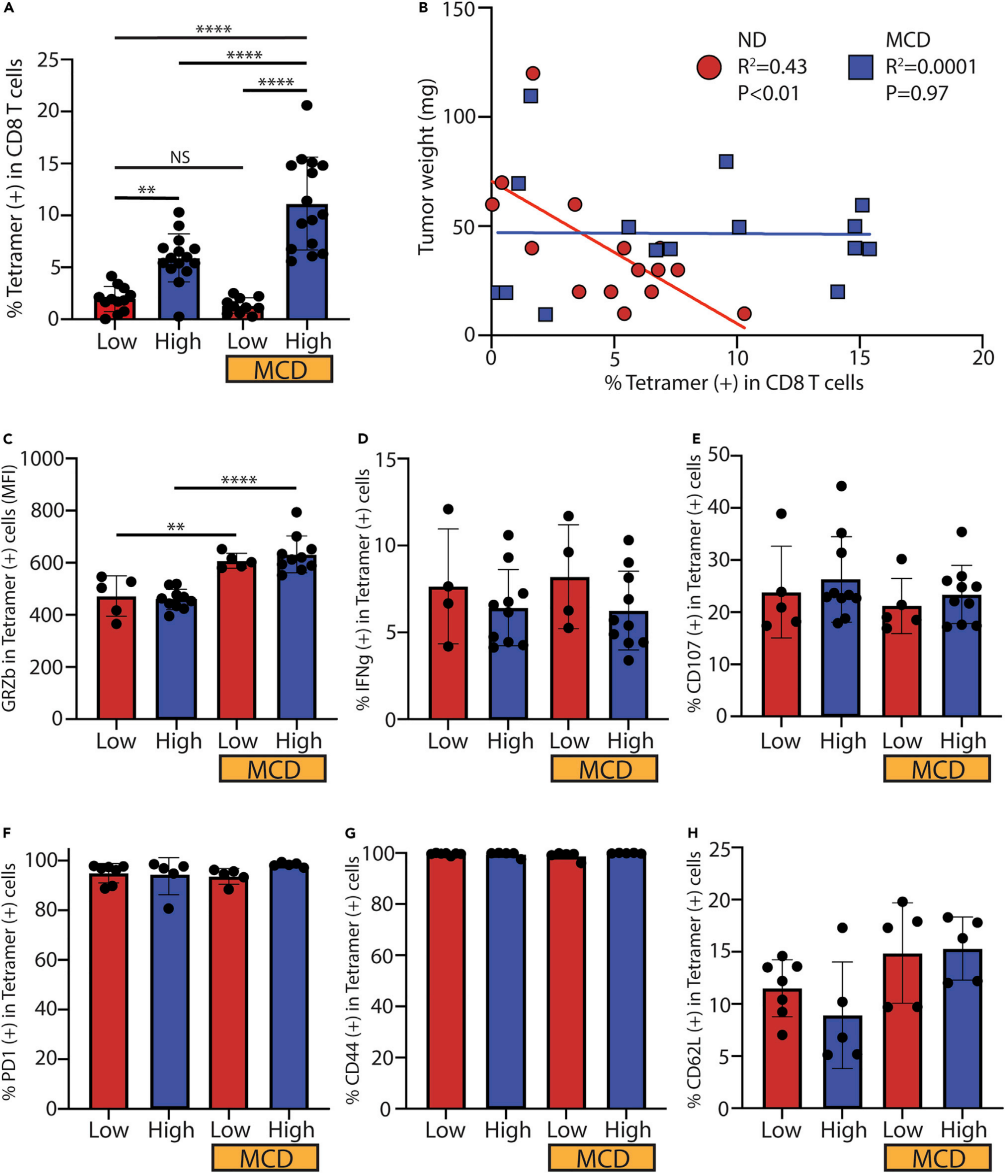
*ex vivo* analysis of OVA_257-264_ tetramer-positive CD8^+^ T cells in normal or NAFLD livers (A–D) Frequency of OVA_257-264_ tetramer-positive CD8^+^ T cells from liver lymphocytes of mice given normal diet (ND) or methionine-choline-deficient (MCD) diet with or without doxycycline (dox). Low means low antigen expression due to no dox water while high means high antigen expression due to dox water. Data pooled from multiple experiments (B) Scatterplot with correlation of tumor weight from ND or MCD diet mice and percentage of tetramer (+) CD8^+^ T cells. Each diet group contains dox on and off. (C) Granzyme B (GRZb) staining of unstimulated OVA tetramer-positive CD8^+^ T cells from liver lymphocytes isolated from ND or MCD mice with or without Dox. (D) IFNg staining in OVA tetramer-positive CD8^+^ T cells after 4 h of stimulation with the OVA_257-264_ peptide. (E) Degranulation assay of liver lymphocytes stimulated with Hep55.1c-Tet3G-LucOS tumor cells incubated with 1ug/mL of Dox. (F) PD-1 staining of unstimulated OVA tetramer-positive CD8^+^ T cells from liver lymphocytes isolated from ND or MCD mice with or without Dox. (G) CD44 staining of OVA tetramer-positive CD8^+^ T cells from liver lymphocytes isolated from ND or MCD mice with or without Dox. (H) CD62L staining of OVA tetramer-positive CD8^+^ T cells from liver lymphocytes isolated from ND or MCD mice with or without Dox. All pooled data presented as mean with SD. One-way ANOVA with Tukey correction for (A) and (C)–(H). ∗p < 0.05; ∗∗p < 0.01; ∗∗∗p < 0.001; ∗∗∗∗p < 0.0001.

Our own results (Figure 1D) and a previous report (Ruiz de Galarreta et al., 2019) show that OVA antigen-induced anti-tumor immunity mostly depends on CD8^+^ T cells. Next, we focused on tumor antigen-specific CD8^+^ T cell response by assessing OVA_257-264_-specific CD8^+^ T cells. As shown before (Figure 1G), a low level of OVA_257-264_-specific CD8^+^ T cells was found in mice bearing liver OVA^low^ tumors, which was not affected by NAFLD (Figure 3A). Consistently, there was an increase in liver OVA_257-264_-specific CD8^+^ T cells in OVA^high^ tumor-bearing mice given normal diet (ND low: 1.95% vs ND high: 5.90%, p < 0.01) (Figure 3A). Unexpectedly, the increase was even more dramatic in NAFLD mice (MCD low: 1.32% vs MCD high: 11.14%, p < 0.0001), and significantly more OVA_257-264_-specific CD8^+^ T cells were found in NAFLD mice as compared to normal diet mice having liver OVA^high^ tumor (ND high: 5.90% vs MCD high: 11.14%, p < 0.0001) (ND high: 11.56% vs Ob high: 20.34%, p < 0.01) (Figures 3A and S2A). The inverse correlation between OVA_257-264_-specific CD8^+^ T cells and tumor weight found in mice given normal diet (R^2^ = 0.43, p < 0.01) was not present in NAFLD mice given MCD diet (R^2^ = 0.0001, p = 0.97) (Figure 3B). Together, these results suggest that NAFLD does not inhibit tumor antigen production or the generation of tumor antigen-specific CD8^+^ T cells, yet these TAS T cells fail to control tumor.

The function of OVA_257-264_-specific CD8^+^ T cells was next tested to determine if there was impairment of TAS CD8^+^ T cells in NAFLD. Intracellular staining revealed that OVA_257-264_-specific CD8^+^ T cells from NAFLD livers had higher levels of the cytotoxic molecule GRZb (Figures 3C and S2B), but similar levels of perforin (Figures S2C and S2E) as compared to normal livers, which was not changed in mice bearing OVA^high^ tumor. Cytokine production of OVA_257-264_-specific CD8^+^ T cells was assessed by measuring IFNg following *ex vivo* OVA_257-264_ peptide stimulation, and similar levels were found across the groups (Figure 3D). There were comparable levels of degranulation in the OVA_257-264_-specific CD8^+^ T cells between NAFLD and normal livers with OVA^low^ or OVA^high^ tumors (Figures 3E and S2D). There were also no differences between PD-1 expression (Figure 3F) as well as other marker of exhaustion including TIM3, LAG3, and CTLA4 (Figure S2F). Finally, effector memory phenotype was also similar as assessed by CD44 (Figure 3G) and CD62L (Figure 3H). These results indicate that in NAFLD, tumor-specific CD8^+^ T cells are not inherently impaired. Thus, the dysfunction of antigen-specific CD8^+^ T cell anti-tumor immunity is likely due to other alterations in the hepatic tumor environment.

### Accumulated macrophages impede the antigen-specific anti-tumor immunity

**Figure 4:**
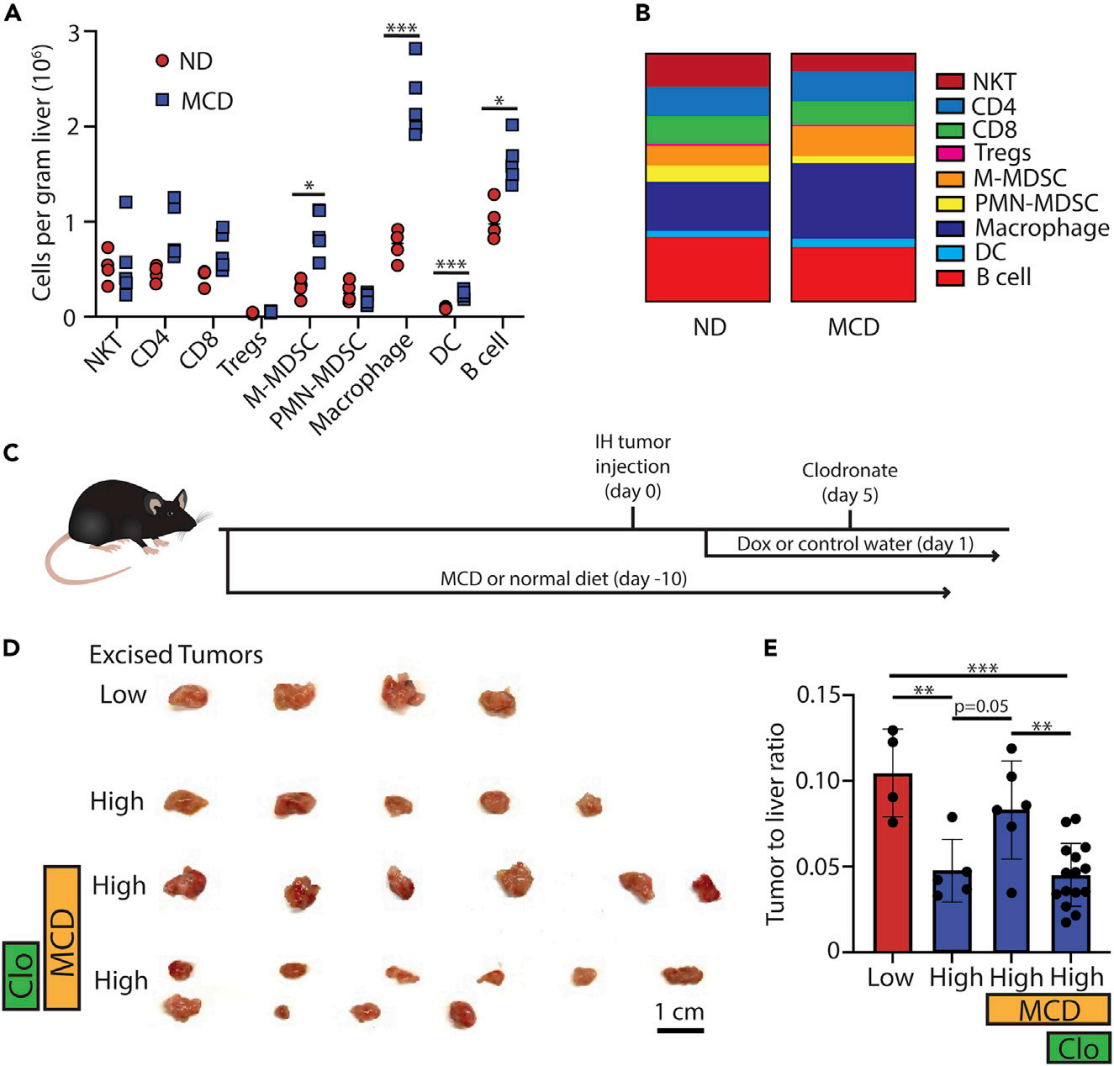
Macrophage suppression of OVA_257-264_-specific CD8^+^ T cells in normal or NAFLD livers. Immune cell monitoring showing (A and B) cells per gram of liver tissue and (B) percentage of live lymphocytes isolated from mice given normal diet (ND) or methionine-choline-deficient (MCD) diet (NKT: natural killer T cell, Treg: T regulatory cell, MDSC: monocytic-myeloid-derived suppressor cell, DC: dendritic cell). (C) Diagram of the experimental design for clodronate depletion. (D and E) Excised tumors and (E) tumor to liver ratio of mice fed ND or an MCD diet with or without dox and clodronate 10 days after an intrahepatic injection of Hep55.1c-Tet3G-LucOS cells. Data pooled from multiple experiments. Low means low antigen expression due to no dox water while high means high antigen expression due to dox water. All pooled data presented as mean with SD. One-way ANOVA with Tukey correction for A and (F) ∗p < 0.05; ∗∗p < 0.01; ∗∗∗p < 0.001; ∗∗∗∗p < 0.0001.

Flow cytometry-based immune profiling was performed on tumor-bearing mice with or without NAFLD to characterize the changes of the liver local immune environment. Minimal changes were observed in the number and percentage of NKT, CD4^+^ T cells, CD8^+^ T cells, T regulatory (Treg), and polymorphonuclear-myeloid-derived suppressor cells (PMN-MDSCs) (Figures 4A and 4B). There were, however, large increases in macrophages, B cells, monocytic-myeloid-derived suppressor cells (M-MDSCs), and dendritic cells (DC) in NAFLD livers (Figures 4A and 4B). Phenotypic analysis of the macrophages between the diets showed that MCD livers had a higher percentage of M2 markers as compared to ND (Figure S2G). The well-documented immunosuppressive function of tumor macrophages (Noy and Jeffrey, 2014) prompted us to study their contribution to the impaired antigen-specific anti-tumor immunity in NAFLD.

Next, macrophages were depleted using clodronate and the impact on liver tumors was tested. Mice were given normal or MCD diet for 10 days and then injected with Hep55.1c-Tet3G-LucOS tumors. OVA^high^ tumor-bearing mice were injected with clodronate (Figures 4C). Similar as before, OVA^high^ tumors were smaller than OVA^low^ tumors in mice given normal diet, and the tumor suppression was reversed in NAFLD mice (ND low: 0.10 vs ND high: 0.05, p < 0.01; ND high: 0.05 vs MCD high: 0.08, p = 0.05). However, depleting macrophages removed the resistance of liver tumor to OVA antigens-induced tumor suppression in NAFLD mice (MCD high: 0.08 vs MCD high + clodronate: 0.05, p < 0.01). In fact, the tumor size was reduced to the similar level compared to OVA^high^ tumors in mice fed with normal diet (Figures 4D and 4E). Similar levels of OVA_257-264_-specific CD8^+^ T cells were found after clodronate treatment in NAFLD mice suggesting that the tumor suppression was not through inducing more tumor-specific CD8^+^ T cells (Figure S2H). Taken together, these results suggest that the impaired antigen-specific CD8^+^ T cell anti-tumor immunity is in part related to the accumulation of macrophages in the local liver immune environment.

## Discussion

To the best of our knowledge, this is the first study to investigate in detail the influence of NAFLD on TAS T cell response. We created an HCC mouse model which can generate a strong inducible anti-tumor T cell response depending on tumor induction of MHC-I OVA_257-264_ and MHC-II _OVA329-337_ antigens. In this model, TAS CD8^+^ T cell response can be monitored by measuring OVA_257-264_-specific CD8^+^ T cells using a tetramer. Using this model, we found that NAFLD impairs anti-tumor CD8^+^ T cell immunity in both an MCD diet model and genetic Ob mice. The generation of TAS CD8^+^ T cells is not inhibited by NAFLD. Importantly, the OVA_257-264_-specific CD8^+^ T cells generated in NAFLD do not show obvious functional impairment, which suggests the impaired anti-tumor immunity is caused by the changed liver immune environment. Indeed, removing the accumulated liver macrophages reversed the NAFLD-caused suppression of antigen-specific anti-tumor inhibition. Our study suggests that critical local environmental changes within NAFLD cause regulation of TAS T cell immunity.

NAFLD is one of the most common causes of HCC in western countries and quickly becoming a significant cause in other parts of the world (Llovet et al., 2021). A growing body of evidence suggests that NAFLD has a significant impact on adaptive T cell immunity which affects liver cancer development and immunotherapy efficacy (Hou et al., 2020). Our group has previously shown that there is a selective loss of CD4^+^ T cells due to a buildup of linoleic acid within mitochondria which impaired the efficacy of CD4^+^ T-dependent tumor vaccination against liver tumors in mice (Ma et al., 2016; Brown et al., 2018a; Heinrich et al., 2021). Other studies using mouse and patient data have shown that NAFLD has large influences on liver CD8^+^ T cells which present a more activated proinflammatory phenotype with higher activation markers and enhanced cytokine production. A recent study identified that livers accumulated autoaggressive CXCR6^+^ CD8^+^ T cells which kills hepatocytes in an MHC-I-independent manner and contribute to NAFLD disease progression. Although it is a consensus that NAFLD causes activation of CD8^+^ T cells, their role in liver tumor development is controversial. In different NAFLD-HCC mouse models, removing CD8^+^ T cells caused opposite effects on HCC progression (Shalapour et al., 2017; Pfister et al., 2021; Wolf et al., 2014). Importantly, immune checkpoint inhibitors, which are believed to act through CD8^+^ T cells, show differential efficacy against HCC in NAFLD. Reduced efficacy of anti-PD-1 therapy was suggested in a meta-analysis of patients with HCC and a CD-HFD mouse HCC model (Pfister et al., 2021). In contrast, anti-PD-L1 therapy effectively reduced HCC in an HFD-fed MUP-uPA mouse model (Shalapour et al., 2017). These studies suggest that further investigations are needed to better understand the role of immune regulation of liver cancer in NAFLD.

TAS T cells are an essential component of the adaptive immune system to mount an immune response to cancer (Chalabi et al., 2020; DuPage et al., 2011, 2012; Flecken et al., 2014; Mandal et al., 2019; Tran et al., 2014; Tumeh et al., 2014). TAS T cells often comprise a small fraction of the total T cells even in tumor-infiltrating lymphocytes. Their changes are often difficult to find in T cell analysis even by using sc-RNA-seq without tetramer staining or identification of TAS TCRs (Caushi et al., 2021). In previous reports, the regulation of TAS T cells in NAFLD has not been carefully studied. Here, using the newly generated mouse model we clearly demonstrate that NAFLD impairs antigen-specific CD8^+^ T cell anti-tumor immunity, and the effect is not CD8^+^ T cell intrinsic but caused by changed liver immune environment such as accumulated macrophages. Our finding suggests a critical influence of NAFLD changed local environment on anti-tumor functionality of CD8^+^ T cells. The finding also helps to explain that the discrepant role of CD8^+^ T cells in NAFLD-promoted HCC was likely due to different liver immune landscape of NAFLD-HCC models. In addition, anti-PD-L1 therapy not only acts on CD8^+^ T cells but also affects other PD-L1 expressing cells. Indeed, a recent study suggests that tumor-associated macrophages are the primary source of PD-L1 in cholangiocarcinoma, and targeting myeloid cells sensitizes cholangiocarcinoma to anti-PD1 therapy (Loeuillard et al., 2020). In addition, HCC mouse models targeting myeloid cells have been found to improve immunotherapy efficacy (Liu et al., 2020). These reports fit well with our result that depleting macrophages reversed the NAFLD-caused resistance to TAS CD8^+^ T cells of Hep55.1c-Tet3G-LucOS tumor. This study suggests that targeting tumor macrophages may benefit immunotherapy for liver cancer patients with NAFLD.

In conclusion, this study investigated the effect that NAFLD had on TAS T cells against HCC. We used a novel inducible OVA-expressing HCC cell line to show that tumor rejection was impaired in two NAFLD mouse models. We found that OVA-specific CD8^+^ T cells from NAFLD mice did not have changes in quantity or function compared to ND mice in an *ex vivo* setting. Immune profiling showed that there was an accumulation of macrophages in NAFLD mice that was not observed in ND mice. Depletion of macrophages with clodronate decreased tumor size suggesting that they impair TAS CD8^+^ T cells in NAFLD.

### Limitations of the study

Several limitations need to be taken into consideration when interpreting the results from this study. This study did not investigate the effects of TAS CD4^+^ T cells because CD8^+^ T cells are the main divers of tumor rejection in this model system. As highlighted by previous papers, CD4^+^ T cells can play an important role in anti-tumor immunity (Alspach et al., 2019; Borst et al., 2018; Kreiter et al., 2015). This model could potentially be modified for investigation of TAS CD4^+^ T cells. Another point that needs to be mentioned is that this study used highly immunogenic chicken OVA as a model antigen instead of real syngeneic tumor antigens. AFP, NY-ESO-1, GPC3, WT1, and other tumor antigens have been identified in HCC (Inada et al., 2019). However, these antigens are more difficult to study and currently lack robust tools to study these TAS T cells. Although highly immunogenic, OVA antigens are well studied, and OVA-expressing tumors are accepted for studying TAS immune response (DuPage et al., 2011, 2012). It should be noted that the Hep55.1c-Tet3G-LucOS tumor is highly immunogenic, but we are still able to observe impaired CD8^+^ T cell anti-tumor immunity, which highlights the strong impact of NAFLD on adaptive anti-tumor immunity.

## STAR★Methods

### Key resources table

**Table:** 

REAGENT or RESOURCE	SOURCE	IDENTIFIER
**Antibodies**		

Mouse anti-CD3-PE	Biolegend	clone 17A2
Mouse anti-CD3-BV650	Biolegend	clone 17A2
Mouse anti-CD107a-FITC	Biolegend	Clone 1D4B
Mouse anti-CD4-BV605	Biolegend	clone RM4-5
Mouse anti-CD4-AF700	Biolegend	clone GK1.5
Mouse anti-CD8-BV421	Biolegend	clone 53-6.7
Mouse anti-CD8-BV785	Biolegend	clone 53-6.7
Mouse anti-Ly6C-APC/Cy7	Biolegend	clone 1A8
Mouse anti-Ly6G-AF700	Biolegend	clone HK1.4
Mouse anti-CD19-PErCp-Cy5.5	Biolegend	clone 6D5
Mouse anti-F4/80-FITC	Biolegend	clone BM8
Mouse anti-F4/80-AF700	Biolegend	clone BM8
Mouse anti-CD11b-BV421	Biolegend	clone M1/70
Mouse anti-CD11b-AF700	Biolegend	clone M1/70
Mouse anti-TCRb-PE/Cy7	Biolegend	clone H57-597
Mouse anti-MHCII-BV510	Biolegend	clone M5/114.15.2
Mouse anti-CD11c-BV650	Biolegend	clone N418
Mouse anti-B220-AF700	Biolegend	clone RA3-6B2
Mouse anti-IFNg-PE	Biolegend	clone XMG1.2
Mouse anti-GranzymeB-FITC	Biolegend	clone GB11
Mouse anti-PD1-BV421	Biolegend	clone 29F.1A12
Mouse anti-TIM3-BV421	Biolegend	clone RMT3-23
Mouse anti-LAG3-APC	Biolegend	clone C9B7W
Mouse anti-CTLA4-PeCy7	Biolegend	clone L3D10
Mouse anti-H-2K^b^-SIINFEKL-PE	Biolegend	clone 25-D1.16
Mouse anti-H-2K^b^-AF647	Biolegend	clone 28-8-6
Mouse anti-CD80	Biolegend	clone 16-10A1
Mouse anti-CD86	Biolegend	clone PO3
Mouse anti-CD163	Biolegend	clone S15049I
Mouse anti-CD206	Biolegend	clone C068C2
Mouse anti-IgG1 isotype control-AF647	Biolegend	clone RMG1-1
Mouse anti-CD4	Biolegend	clone GK1.5
Mouse anti-CD8	BD Horizon	clone 53-6.7

**Bacterial and virus strains**		

NEB 5-alpha Competent *E. coli*	New England Biolabs	Cat# C29871

**Chemicals, peptides, and recombinant proteins**		

Human recombinant IFNg	Invivogen	Cat# rcyec-hifng
SIINFEKL (OVA_257-264_ peptide)	Invivogen	Cat# vac-sin
Golgistop	BD biosciences	Cat# BD554724
Golgiplug	BD biosciences	Cat# BD555029
1x PBS	Corning	Cat# 21-040-CMX12
RPMI1640 media	Corning	Cat# 10-040-CV
GlutaMAX	ThermoFisher	Cat# 35050061
Tetracycline free FCS	Takara Bio	Cat# 631106
Penicillin/Streptomycin	ThermoFisher	Cat# 15140122
Puromycin	ThermoFisher	Cat# A1113802
Neomycin	ThermoFisher	Cat# 21810031
Doxycycline	Sigma Aldrich	Cat# 324385
Methionine choline deficient	Research diet	Cat# A02082002BRi
Matrigel	Corning	Cat# 354234
Nde1	New England Biolabs	Cat# R0111S
Apa1	New England Biolabs	Cat# R0114S

**Critical commercial assays**		

Lenti-X Tet-On 3G Inducible Expression System	Takara Bio	Cat# 631187
Lenti-X Packaging Single Shots	Takara Bio	Cat# 631278
Promega Luciferase Assay kit	Promega	Cat# E6110
MiniPrep kit	Qiagon	Cat# 27106X4
Foxp3/transcription factor staining kit	eBiosciences	Cat# 00-5523-00
Live/dead near-IR dead cell staining kit	ThermoFisher	Cat# L10119
Zombie UV fixable dead cell staining kit	Biolegend	Cat# 423107

**Experimental models: Cell lines**		

Hep55.1c mouse HCC cell line	CLS	Cat# 400201
Hek293T cells	ATCC	Cat# CRL-3216

**Experimental models: Organisms/strains**		

C57BL/6	Jackson Laboratory	Stock# 000664
C57BL/6-Tg(TcraTcrb)1100Mjb/J	Jackson Laboratory	Stock# 003831
B6.Cg-Lep^ob^/J	Jackson Laboratory	Stock# 000632

**Oligonucleotides**		

LucOS forward primer	Integrated DNA Tech	1030615972
LucOS reverse primer	Integrated DNA Tech	1030603510

**Recombinant DNA**		

Lenti-LucOS	Addgene	Plasmid# 22777

**Software and algorithms**		

Prism 9	GraphPad Software	Version 9.1.2
FlowJo software version 10.4.2	Becton Dickinson & co	Version 10.7.2
Adobe Illustrator	Adobe	Version 26.0.1

**Other**		

CD1d-APC tetramer	Emory Tetramer Core	TO# 54407
H-2K^b^-SIINFEKL-PE tetramer	Emory Tetramer Core	TO# 49278

### Resource availability

#### Lead contact

Further information and requests for resources and reagents should be directed to and will be fulfilled by the lead contact, Dr. Chi Ma (chi.ma2@nih.gov).

#### Materials availability

• Plasmids generated in this study are available upon request of reagent from lead contact.
• Cell lines generated in this study are available upon request of reagent from lead contact.
• There were no new mouse lines generated in this manuscript.
• This study did not generate new unique reagents.

### Experimental model and subject details

#### Cell culture

Hep55.1c mouse HCC cell line was purchased from Cell Lines Service (CLS, Germany). Hek293T cells were purchased from ATCC (ATCC, Manassas VA). The cells were maintained in RPMI1640 media supplemented with GlutaMAX, 5% tetracycline free FCS, and 1% penicillin/streptomycin. The cells were kept in an incubator at 37°C and 5% CO_2_. Cells were passaged after they had reached 70% confluency and transferred to a new flask in a 1:10 or 1:20 ratio. Lentivirus transduction to create the Hep55.1c-Tet3G-LucOS cell line was done following the Takara Bio protocol. Selection for positively transduced cells was done with Puromycin and Neomycin.

#### Animal studies

Female 6–10 weeks old C57BL/6 (Black6) (The Jackson Laboratory, Bar Harbor ME, stock no 000058), C57BL/6-Tg(TcraTcrb)1100Mjb/J mice (OT-I) (The Jackson Laboratory, Bar Harbor ME, stock no 003831), or B6.Cg-Lep^ob^/J mice (Ob) (The Jackson Laboratory, Bar Harbor ME, stock no 000632) were utilized for mouse experiments. Mice were randomized for all experiments. NAFLD was induced by feeding mice a methionine choline deficient (MCD) diet for 10-14 days (Research Diets, New Brunswick NJ) or once Ob mice had reached 12 weeks on standard chow. Mice were kept on their specific diet until experimental endpoints. 2 mg/mL of doxycycline with 10% glucose (Sigma Aldrich, St. Louis MO) of drinking water was added where indicated. Drinking water was replaced every five days. Intrahepatic tumor injections were performed 10 to 14 days after the start of the MCD diet or once Ob/Ob mice were 12 weeks old. A mouse model using intrahepatic injection has been described in detail elsewhere (Brown et al., 2018b). For intrahepatic tumors, 20μL of 2x10^5^ Hep55.1c-Tet3G-LucOS cells in 1:1 1x PBS and Matrigel (Corning, NY) were injected under the capsule of the left liver lobe of anesthetized mice. Results were presented as tumor weight (mg) or taking the tumor weight and dividing it by the liver weight (tumor to liver ratio) to account for differences in animal weight induced by the diet. Some tumor samples were submitted for H&E staining. Macrophage depletion was done by injecting clodronate liposomes into the tail vein of mice on day 5 after intrahepatic injection. To establish subcutaneous tumors, 1x10^6^ cells were re-suspended in 1x PBS and then injected in the right lateral flank. Depletion of CD4 (Biolegend) and CD8 (BD Horizon) T cells were conducted by intraperitoneal injection of 200ug of antibody every 7 days starting on the day before injection. Tumor size was measured by calculating the longest diameter using calipers. Isolation of liver lymphocytes from tumor-bearing mice has been described previously (Kapanadze et al., 2013). All experiments were conducted according to local institutional guidelines and approved by the Animal Care and Use Committee of the National Institutes of Health, Bethesda, USA.

### Method details

#### Plasmid construction

The Lenti-X Tet-On 3G tetracycline-inducible expression system is commercially available and purchased through Takara Bio (Takara Bio USA Inc, Shiga, Japan). The Lenti-LucOS plasmid was a gift from Tyler Jacks laboratory (Addgene plasmid #22777). The LucOS gene contains the luciferase gene conjugated to two chicken ovalbumin antigens (MHC I and II restricted) as well as a synthetic MHCI antigen. These antigens are not endogenously produced by tumors but have been used previously to study T cell biology in HCC making them a well-accepted model. The LucOS gene was amplified using a forward primer containing the *Apa*I restriction enzyme site (TAGCTGGGCCCATGGAAGACGCCAAAAACATAAAG) and reverse primer containing the *Nde*I restriction enzyme site (TCGATGCATATGTTAC AAGTCCTCTTCAGAAATAAGC). After amplification of the LucOS gene, the product and the Lenti-Tre3G plasmid were digested with *Apa*I and *Nde*I (New England Biolabs, Ipswich MA). The two digested products were then ligated together to create a Lenti-Tre3G-LucOS plasmid. Transformation was done using DH5alpha competent cells (New England Biolabs, Ipswich MA). Plasmid expansion was then done using Qiagen Miniprep kit (Qiagen, Hilden Germany).

#### Luciferase assay

Cells were plated out in a 24 well plate at a concentration of 1 x 10^5^ in 1mL of media. Increasing concentrations of doxycycline was then added in triplicates. Twenty-four hours later, the media was aspirated, and cells were washed with 2mL of 1x PBS. A luciferase reporter assay was preformed using the Promega Luciferase Assay kit (Promega, Madison WI). Luminescence was assessed using a Bio-Rad plate reader (Bio-Rad, Hercules CA).

#### T-cell stimulation assay

Hep55.1c-Tet3G-LucOS cells were plated in a 6-well plate at 5 × 10^5^ in 2 mL of media. Desired concentrations of doxycycline and/or 100ng of human recombinant IFNg (Invivogen, San Diego CA) were added to the media. The cells were allowed to incubate overnight. The next day, splenocytes from an OT-I mouse were isolated and 2.5 x 10^6^ cells were added to each well. Positive controls (PC) were isolated OT-I splenocytes pulsed overnight with 10ng of OVA_257-264_ peptide (Invivogen, San Diego CA). Negative controls (NC) were un-transduced Hep55.1c cells that did not express LucOS. 3uL of Golgiplug and 2uL of Golgistop were then added to each well containing OT-I cells (BD biosciences, East Rutherford NJ). The OT-I tumor cell mixture was then incubated for 4-6 h at 37°C at 5% CO_2_.

#### Degranulation assay

Hep55.1c-Tet3G-LucOS cells were incubated over night with 1ug/mL of dox and 10 ng/mL of IFNg. The next day, 100uL of 5 × 10^4^ tumor cells in complete media supplemented with 0.7uL/mL of GolgiStop and 1/200 dilution CD107a-FITC (clone 1D4B, Biolegend) antibody were added to a 96 well plate. 100uL of 1 × 10^6^ liver lymphocytes isolated from tumor bearing mice were then added to the mixture for a total of 200uL per well. The lymphocyte tumor mixture was incubated for 5 h at 37^°^C and 5% CO_2_.

#### Flow cytometric analysis

Cells were incubated with the indicated antibodies or tetramer for 15 min at 4°C. Foxp3/transcription factor staining buffer set (eBioscience) was used for intracellular staining according to the manufacturer’ instructions. Intracellular antibody staining was done for 1 h at 4°C. Flow cytometry was performed on a Beckman Coulter Cytoflex LX platform and results were analyzed using FlowJo software version 10.4.2 (TreeStar). Dead cells were excluded by using live/dead near-IR or zombie UV fixable dead cell staining kit (ThermoFisher scientific or Biolegend). The following antibodies were used for flow cytometry analysis (all anti-mouse): CD3-PE (clone 17A2, Biolegend), CD3-BV650 (clone 17A2, Biolegend), CD4-BV605 (clone RM4-5, Biolegend), CD4-AF700 (clone GK1.5, Biolegend), CD8-BV421 (clone 53-6.7, Biolegend), CD8-BV785 (clone 53-6.7, Biolegend), Ly6G-AF700 (clone 1A8, Biolegend), Ly6C-APC/Cy7 (clone HK1.4, Biolegend), CD1d-APC tetramer (Emory tetramer core), CD19-PErCp-Cy5.5 (clone 6D5, Biolegend), F4/80-FITC (clone BM8, Biolegend), F4/80-AF700 (clone BM8, Biolegend), CD11b-BV421 (clone M1/70, Biolegend), CD11b-AF700 (clone M1/70, Biolegend), TCRb-PE/Cy7 (clone H57-597, Biolegend), MHCII-BV510 (clone M5/114.15.2, Biolegend), CD11c-BV650 (clone N418, Biolegend), B220-AF700 (clone RA3-6B2, Biolegend) IFNg-PE (clone XMG1.2, Biolegend), GranzymeB-FITC (clone GB11, Biolegend), PD1-BV421 (clone 29F.1A12, Biolegend), TIM3 (clone RMT3-23, Biolegend), LAG3 (clone C9B7W, Biolegend), CTLA4 (clone L3D10, Biolegend), CD80 (clone 16-10A1, Biolegend), CD86 (clone PO3, Biolegend), CD163 (clone S15049I, Biolegend), CD206 (clone C068C2, Biolegend), H-2K^b^-SIINFEKL-PE (clone 25-D1.16, Biolegend), H-2K^b^-SIINFEKL-PE tetramer (Emory tetramer core), H-2K^b^-AF647 (clone 28-eight to six, Biolegend), and anti-IgG1 isotype control-AF647 (clone RMG1-1, Biolegend).

### Quantification and statistical analysis

All data is presented as means with SD. Means between treatment groups were compared using two-tailed unpaired T-test or one-way ANOVA. Multiple comparisons were accounted for by using the Bonferroni-Dunn method in the case of T-test or the Tukey method for one-way ANOVA. Correlations were determined using simple linear regression and presented as Pearson correlation coefficient (R^2^ values). Specific details regarding each experiment are in the figure legends. All analyses were carried out using GraphPad Prism 9.0.1. Significance is denoted as follows: not significant (ns) p> 0.05, ∗p < 0.05, ∗∗p < 0.01, ∗∗∗p < 0.001 and ∗∗∗∗p < 0.0001.

## Data Availability

• All data reported in this paper will be shared by the lead contact upon request. • This paper does not report original code. • Any additional information required to reanalyze the data reported in this paper is available from the lead contact upon request.
