# Supplementary material for: NAFLD indirectly impairs antigen-specific CD8+ T cell immunity against liver cancer in mice
Source: iScience. 2022 Feb 1;25(2):103847. doi: 10.1016/j.isci.2022.103847 (PMC8844694; doi:10.1016/j.isci.2022.103847)
Supplement: Document S1. Figure S1 and S2 [file mmc1.pdf]

iScience, Volume *25*

Supplemental information

NAFLD indirectly impairs antigen-specific $CD8^+$ T cell immunity against liver cancer in mice

John C. McVey, Benjamin L. Green, Benjamin Ruf, Justin D. McCallen, Simon Wabitsch, Varun Subramanyam, Laurence P. Diggs, Bernd Heinrich, Tim F. Greten, and Chi Ma

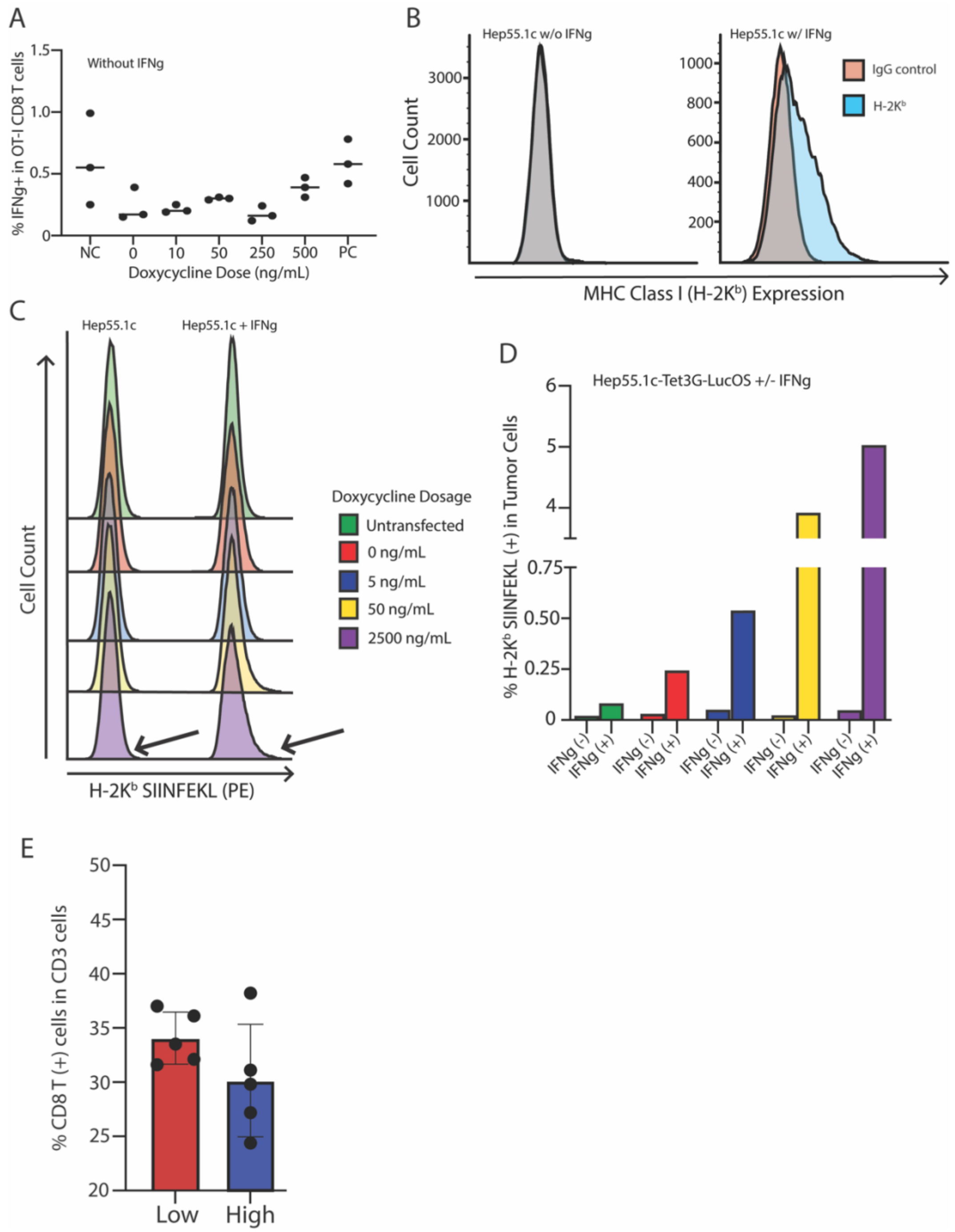

Figure S1: Characterization of the Hep55.1c-Tet3G-LucOS cell line, related to figure 1. (A) Intracellular IFNg production in OT-I CD8+ T cells co-incubated with Hep55.1c-Tet3G-LucOS cells as well as increasing concentrations of doxycycline. Negative control (NC) was untransduced Hep55.1c cells and positive control (PC) was Hep55.1c cells pulsed with $OVA_{257-264}$ peptide. **(B)** H-2K^b expression compared to IgG control of Hep55.1c-Tet3G-LucOS cells with or without IFNg (10ng/mL). **(C and D)** Anti-H-2K^b-SIINFEKL of Hep55.1c-Tet3G-LucOS cells with increasing doxycycline concentration and with or without IFNg (10ng/mL). **(E)** CD8+ T cells from liver lymphocytes of mice given control or doxycycline water. Low means low antigen expression due to no dox water while high means high antigen expression due to dox water. All pooled data presented as mean with standard deviation. One-way ANOVA with Tukey correction for A and E. *$p<0.05$; **$p<0.01$; ***$p<0.001$; ****$p<0.0001$

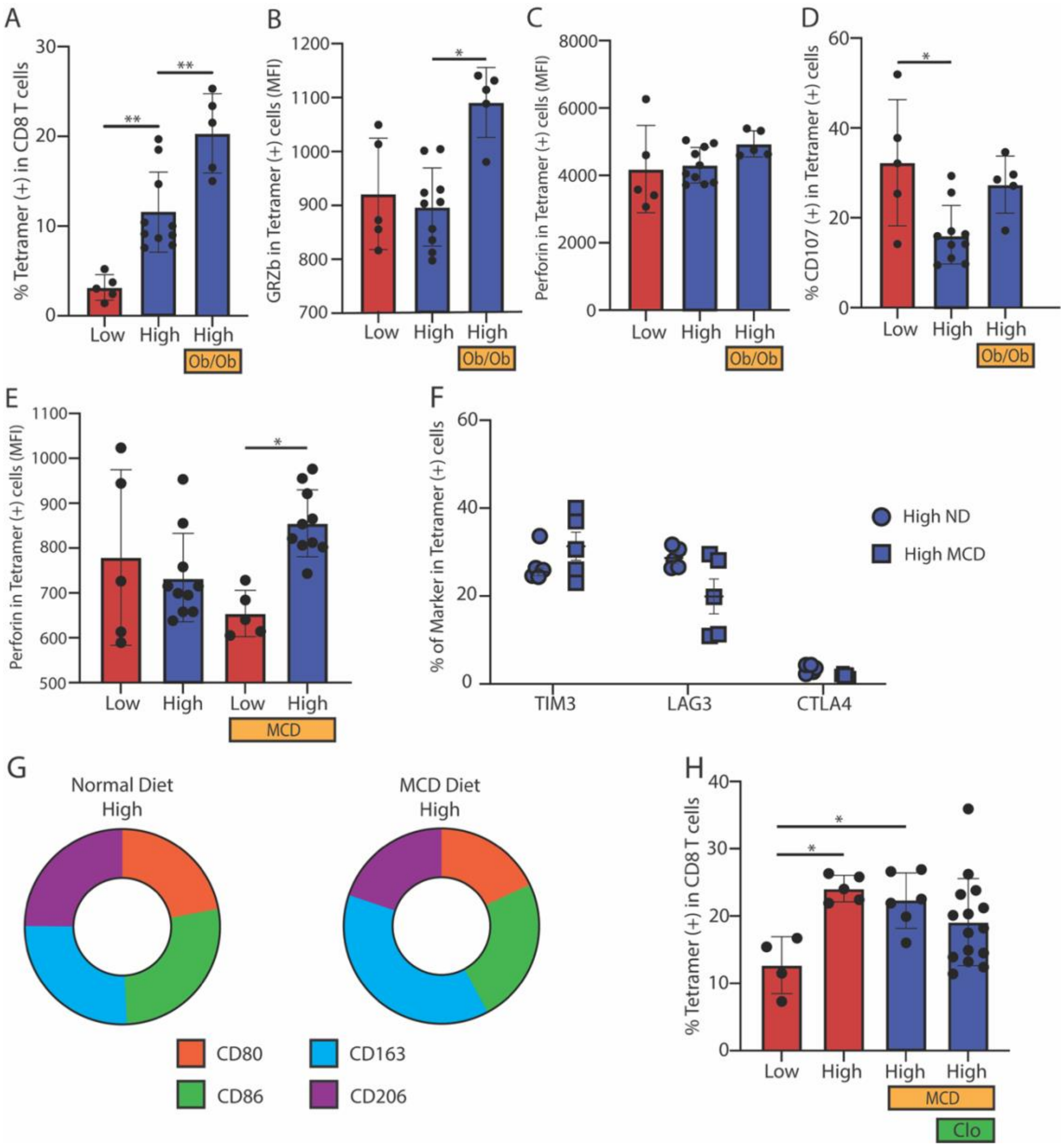

Figure S2: Further characterization of OVA$_{257\text{-}264}$ MHC-I tetramer positive CD8^{+} T cells in normal or NAFLD livers, related to figure 3 and 4. (A) OVA$_{257\text{-}264}$ tetramer positive CD8^{+} T cells from liver lymphocytes of C57BL/6 WT or Ob/Ob (Ob) mice with or without Dox. Low means low antigen expression due to no dox water while high means high antigen expression due to dox water. **(B)** Granzyme B (GRZb) staining of unstimulated OVA tetramer positive CD8^{+} T cells from liver lymphocytes isolated from C57BL/6 or Ob mice with or without Dox. **(C)** Perforin staining of unstimulated OVA tetramer positive CD8^{+} T cells from liver lymphocytes isolated from C57BL/6 or Ob mice with or without Dox. **(D)** Perforin staining of unstimulated OVA tetramer positive CD8^{+} T cells from liver lymphocytes isolated from ND or MCD mice with or without Dox. **(E)** Degranulation assay of liver lymphocytes from C57BL/6 or Ob mice stimulated with Hep55.1c-Tet3G-LucOS tumor cells incubated with 1ug of Dox. **(F)** Markers of exhaustion including TIM3, LAG3 and CTLA4 of mice treated with ND or MCD and with Dox **(G)** Characterization of M1 vs M2 macrophages isolated from C57BL/6 ND or MCD mice given doxycycline water. **(H)** OVA$_{257\text{-}264}$ tetramer positive CD8^{+} T cells from liver lymphocytes of mice given ND or MCD diet with or without dox or clodronate. All pooled data presented as mean with standard deviation. One-way ANOVA with Tukey correction for A-F. *p<0.05; **p<0.01; ***p<0.001; ****p<0.0001.
